# Prognostic value of tertiary lymphoid structures (TLS) in digestive system cancers: a systematic review and meta-analysis

**DOI:** 10.1186/s12885-023-11738-w

**Published:** 2023-12-18

**Authors:** Hao Sun, Yuanyu Shi, Hailiang Ran, Junwei Peng, Qiongxian Li, Guiqing Zheng, Yandie He, Shuqing Liu, Wei Chang, Yuanyuan Xiao

**Affiliations:** NHC Key Laboratory of Drug Addiction Medicine, Division of Epidemiology and Health Statistics, School of Public Health, Kunming Medical University, Chengong District, 1168 West Chunrong Road, Yuhua Street, Kunming, Yunnan China

**Keywords:** Tertiary lymphatic structure (TLS), Digestive system cancers, Prognosis, Meta-analysis

## Abstract

**Background:**

Existing literature suggests that tertiary lymphatic structure (TLS) is associated with the progression of cancer. However, the prognostic role of TLS in digestive system cancers remains controversial. This meta-analysis aims to synthesize currently available evidence in the association between TLS and the survival of digestive system cancers.

**Methods:**

We systematically searched three digital databases (PubMed, Embase, Web of Science) for articles published from database inception to December 23, 2022. Study selection criteria are based on PECO framework: P (population: patients with digestive system cancers), E (exposure: presence of TLS), C (comparator: absence of TLS), O (outcome: overall survival, OS; recurrence-free survival, RFS; disease-free survival, DFS). The Quality in Prognostic Studies (QUIPS) tool was used to assess risk of bias for included studies. The study protocol was registered with PROSPERO (CRD42023416307).

**Results:**

A total of 25 studies with 6910 patients were included into the final meta-analysis. Random-effects models revealed that the absence of TLS was associated with compromised OS, RFS, and DFS of digestive system cancers, with pooled hazard ratios (HRs) of 1.74 (95% CI: 1.50–2.03), 1.96 (95% CI: 1.58–2.44), and 1.81 (95% CI: 1.49–2.19), respectively. Subgroup analyses disclosed a stronger TLS-survival association for pancreatic cancer, compared with other digestive system cancers.

**Conclusion:**

TLS may be of prognostic significance for digestive system cancers. More original studies are needed to further corroborate this finding.

**Supplementary Information:**

The online version contains supplementary material available at 10.1186/s12885-023-11738-w.

## Background

Digestive system cancers, including esophageal carcinoma (EC), gastric cancer (GC), colorectal cancer (CRC), hepatocellular carcinoma (HCC), pancreatic cancer (PC), are leading causes of global cancer-related morbidity and mortality. CRC, GC, HCC, and EC take 4 places in the top 10 cancers by incidence [1–4]. Three out of the top five global cancer-related mortality can be ascribed to digestive system cancers [1]. In addition to high morbidity and mortality, the prognosis of digestive system tumors are not optimistic, with overall 5-year survival rates of 11.5%, 20.8%, and 33.3% for PC, HCC, and GC in the US from 2012 to 2018 [5–7]. Exploring meaningful prognostic markers of digestive system cancers are vital for clinical treatment of the patients.

Tertiary lymphoid structure (TLS) is defined as ectopic lymphocyte aggregates in non-lymphoid tissues when chronic inflammation like tumors, autoimmune diseases, and chronic infections arise after birth [8]. TLS includes a T-cell-rich zone containing dendritic cells (DCs) and a B-cell-rich zone containing germinal centers (GCs), surrounded by plasma cells, various lymphocytes freely pass through high endothelial venules (HEVs) [9, 10]. In function, cellular composition, and organization, TLS is similar to secondary lymphoid organs (SLOs). The concept of TLS was first proposed in 1990s [11], in subsequent studies, it has also been referred to as ectopic lymphoid structures (ELS) or tertiary lymphoid organ (TLO) [12, 13].

Controversies exist in the role of TLS in cancer progression. For instance, one study reported that regulatory T cells in tumor-associated TLS can suppress the endogenous immune response against tumors in a genetically engineered mouse model of lung adenocarcinoma [14], another study revealed that TLS formation reduced ovarian tumors growth in mouse model [15]. In recent years, some scholars have begun to investigate the prognostic significance of TLS in cancer patients, and the presence of TLS was found to be associated with a better prognosis in melanoma [16], breast cancer [17], and lung cancer [18]. However, fewer studies on this topic were related to digestive system tumors, with incongruent results [19–22].

Considering existing inconsistencies in the association between TLS and the survival of digestive system cancers, we aim to perform a systematic review and meta-analysis to synthesize currently available evidence.

## Methods

### Search strategy

This study was performed according to the Preferred Reporting Items for Systematic Evaluation and Meta-Analysis (PRISMA) statement guidelines [23]. We used the PECO [24] framework to clearly frame our study topic: P (population: patients with digestive system cancers), E (exposure: presence of TLS), C (comparator: absence of TLS), O (outcome: overall survival, OS; recurrence-free survival, RFS; disease-free survival, DFS). The study protocol was registered with PROSPERO (CRD42023416307).

We systematically searched three digital databases (PubMed, Embase, Web of Science) for articles published from database inception to December 23, 2022. According to our research theme, the keywords used for searching are closely related to “Tertiary Lymphoid Structure”, “digestive system”, “cancer”, and “prognosis”. A detailed search strategy is presented in the supplementary material (Page 2). This search resulted in an initial check of the titles and abstracts of the articles, followed by full-text review, manual inspection of the reference lists of all relevant papers were also performed to ensure no pertinent studies were missed according to the above strategy.

### Inclusion and exclusion criteria

Eligible studies have to meet the following inclusion criteria: (1) Focused on patients with TLS expression in digestive system cancers; (2) TLS was measured according to standard methods; (3) Primary outcome of interest was OS, or RFS, or DFS; (4) Reported complete pathological staging information. Exclusion criteria are as follows: (1) Case reports, animal trials, reviews, or conference abstracts; (2) Investigated TLS in peritumoral tissues; (3) Did not report hazard ratio (HR) or its 95% confidence interval (CI); (4) Focused on only part of the TLS (immune cells, high endothelial vein, etc.) rather than the whole TLS; (5) Overlapping study subjects; (6) Studies published not in English.

### Data extraction and quality evaluation

A standard data extraction form has been designed, two investigators (HS, YS) independently extracted the following information from the included studies: first author, year of publication, country of origin, cancer type/site, sample size, disease stage, laboratory methods, enrollment period of patients, follow-up time, criteria or cut-offs for determining TLS, outcome indicators, HR and 95% CI, presence of metastases. For studies reported both univariate and multivariate results, we extracted multivariate results, for studies only reported univariate results, we extracted univariate results, univariate and multivariate results were combined separately.

We used the Quality in Prognostic Studies (QUIPS) tool to assess risk of bias for included studies [25]. The QUIPS tool consists of six bias domains: study participation, study attrition, prognostic factor measurement, outcome measurement, study confounding, and statistical analysis and reporting. There are 3 to 7 different items for each bias domain. The risk of bias for a single study can be rated as low, moderate, or high.

### Statistical analysis

Associations between TLS expression and the prognosis of digestive system cancers were evaluated by using pooled HRs from random-effects or fixed-effects models. The *I*^2^ was used to assess heterogeneity, usually *I*^2^ > 50% and *p* < 0.05 indicates substantial level of heterogeneity [26]. Sensitivity analyses were performed to test robustness of the combined estimations. Subgroup analysis was performed to estimate heterogeneity introduced by origin of study (China vs. other countries), sample size (< 200 vs. ≥ 200), metastases (yes vs. no), tumor types (ESCC vs. GC vs. CRC vs. HCC vs. PC), and cut-off criteria (presence vs. absence, high vs. low). Funnel plots, Egger’s [27] regression asymmetry test, and Begg’s [28] rank correlation test were used to examine potential publication bias. We use Endnote X9 to filter articles, all statistical analyses were performed using the R software (version 4.2.3), mainly “meta” and “forestploter” packages.

## Results

### Study selection

**Fig. 1:**
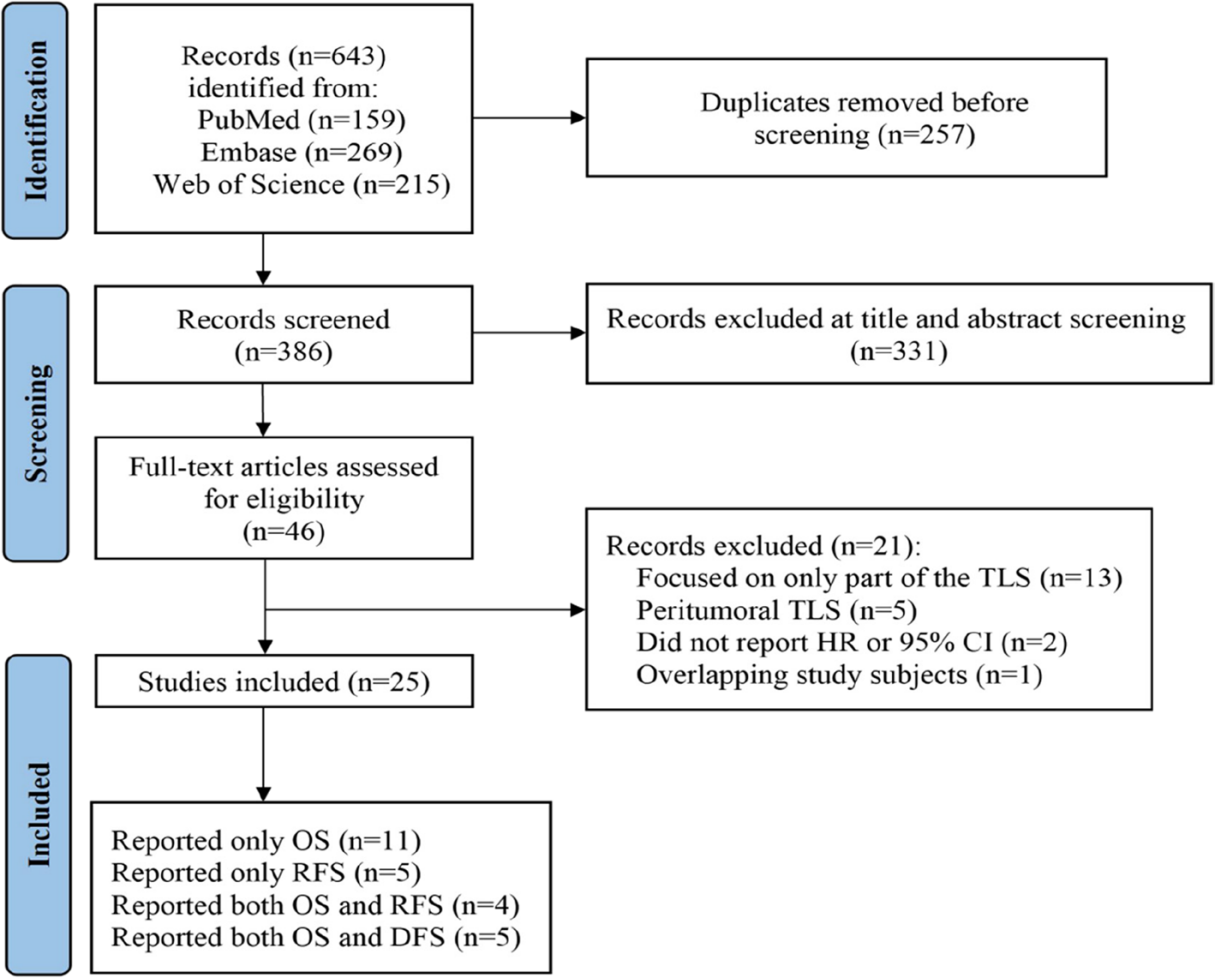
PRISMA flowchart for literature search and study selection

The literature screening process is shown in the PRISMA flowchart presented in Fig. 1. Starting with a total of 643 articles identified from the three databases based on the search strategy, after removing duplicate records, 257 articles remained. After browsing titles and abstracts, we screened out 46 studies that met the inclusion criteria, 21 were further excluded after careful full-text review because of disqualification. Finally, a total of 25 articles were included in the meta-analysis [20, 22, 29–51], including 20 articles reported OS, 9 articles reported RFS, and 5 articles reported DFS.

### Characteristics of included studies

The risk of bias for all finally included studies in this meta-analysis is moderate or low (see in supplementary material, Table S1). The included studies were all retrospective in nature, their major characteristics were shown in Table 1: most studies (19 in 25) were published in 2020 and after; 19 studies were from Asian countries (China: 14, Japan: 5), 3 studies were from Europe (Finland: 1, France: 1, Italy: 1), 2 studies were from Oceania (Austria), and 1 study was from North America (United States); as to specific types of cancer, 4 studies investigated EC, 8 on GC, 4 on CRC, 5 on HCC, and 4 on PC; sample size ranged from 47 (Shota K, 2019, Japan) to 914 (He WT, 2020, China). The criteria for defining TLS were not identical among included studies, most studies used the HE&IHC methods to determine the presence or absence of TLS, and some studies used the number or density of TLS in the tumor to determine high or low distribution of TLS.

**Table 1:** Characteristics of included studies

Author	Year	Country	Types	Patients(n)	Stage	Enrollment period	Follow-up time	Laboratory	Estimate TLS criteria	Cut-off criteria	Metastases	Outcomes
Deguchi S [29]	2022	Japan	ESCC	236	0-IVa	2014	median:51.0 month	HE、IHC	GC-TLS density	High vs. Low	No	RFS
Li RT [30]	2022	China	ESCC	122	I-IV	2017.1–2018.12	More than 2 years	HE、IHC	TLS	Presence vs. Absence	No	OS, DFS
Ling YH [31]	2022	China	ESCC	394	I-III	2008–2017	Median:68.57 month	HE、IHC	TLS	Presence vs. Absence	No	OS, DFS
Jiang Q [32]	2022	China	GC	292	I-IV	1983–2016	Until 2020.1	HE、IHC	Mature TLS	High vs. Low	Yes	OS
Kemi N [33]	2022	Finland	GC	721	I-IV	2002–2008	Median: 28.0 month	HE	TLS diameter	High vs. Low	NA	OS
Zhan Z [34]	2022	China	CRC	203	I-IV	2014.01–2017.07	Median: 50.0 month	IHC	TLS	High vs. None or Low	Yes	OS, DFS
Li JH [35]	2022	China	HCC	150	I-IV	2014–2018	Until 2020.12.30	HE、IHC、IF	TLS	Positive vs. Negative	Yes	RFS
Nie Y [36]	2022	China	HCC	145	0-C	2015–2017	More than 2 years	HE、IHC	TLS Grade	GradeB + C vs. Absence	Yes	OS
Wen SD [37]	2022	China	HCC	126	A-C	2013.04–2019.08	Median: 40.0 month	HE、IHC、IF	TLS density	High vs. Low	Yes	OS
Yu JS [38]	2022	China	GC	118	I-III	2009–2014	Until 2019.05	IHC	TLS	High vs. Low	No	OS, DFS
Cheng N [39]	2021	China	GC	846	I-IV	2008.12–2019.06	Mean: 22.1 month	HE、IHC	TLS	Absent vs. Present	NA	OS
Yamakoshi Y [40]	2021	Japan	GC	199	Ib-IV	2006–2008	Median: 49.0 month	IHC	TLS (B cell density)	Low vs. High	No	OS
Gunderson AJ [41]	2021	America	PC	63	I-III	NA	NA	HE、IHC	TLS	Presence vs. Absence	No	OS
Mori T [42]	2021	Japan	GC	261	I-IV	2014–2017	NA	IHC	TLS	High vs. Low	Yes	OS
Zhao YY [43]	2020	China	ESCC	593	T1	2009–2018	Median: 42.0 month	HE	TLS	Presence vs. Absence	No	OS
He WT [44]	2020	China	GC	914	I-III	2009–2014	Until 2018.1	HE	TLS-SUM	High vs. Low	No	OS
Li Q [45]	2020	China	GC	63	I-III	2001.01–2013.12	Until 2011.11.30	HE	Number of TLS	High vs. Low	No	OS
Li H [46]	2020	China	HCC	303	I-IV	2009.03–2013.8	Median: 61.3 month	HE、IHC	TLS	Presence vs. Absence	Yes	OS, RFS
Zhang WH [47]	2020	China	PC	182	I-III	2006–2018	Median: 60.0 month	HE、IHC	TLS	Presence vs. Absence	No	OS, RFS
Shota K [48]	2019	Japan	PC	47	0-IV	2009.1–2015.12	Median:24.98 month	IHC	TLO density	High vs. Low	Yes	OS
Calderaro J [22]	2018	France	HCC	273	B-C	1995–2016	2 years after surgery	HE	TLS	Presence vs. Absence	Yes	RFS
Posch F [20]	2017	Austria	CRC	109	II-III	1996.01–2011.06	3 years	IF	Number of TLS	< Q1 vs. ≥ Q1	No	RFS
Schweiger T [49]	2016	Austria	CRC	57	I-IV	2009.04–2014.06	Median: 30.0 month	IHC	TLS	Presence vs. Not present	Yes	OS, RFS
Hiraoka N [50]	2015	Japan	PC	308	Ia-IV	1990–2004	Median: 17.6 month	HE、IHC	TLO	Absence vs. Presence	Yes	OS, DFS
Di Caro G [51]	2014	Italy	CRC	185	II-III	1997.01–2005.11	4.71 years	IHC	TLT	< Median vs. ≥ Median	No	RFS

*Abbreviations*: *ESCC* esophageal squamous cell carcinoma, *CRC* colorectal cancer, *GC* gastric cancer, *HCC* hepatocellular carcinoma, *PC* pancreatic cancer, *HE* hematoxylin eosin staining, *IHC* immunohistochemistry, *IF* immunofluorescence, *OS* overall survival, *RFS* relapse-free survival, *DFS* disease-free survival, *TLS* tertiary lymphoid structure, *iTLS* intratumoral tertiary lymphoid structure, *TLO* tertiary lymphoid organ, *GC-TLS* germinal center tertiary lymphoid structure, *NA* not available

### TLS with OS of digestive system cancers

**Fig. 2:**
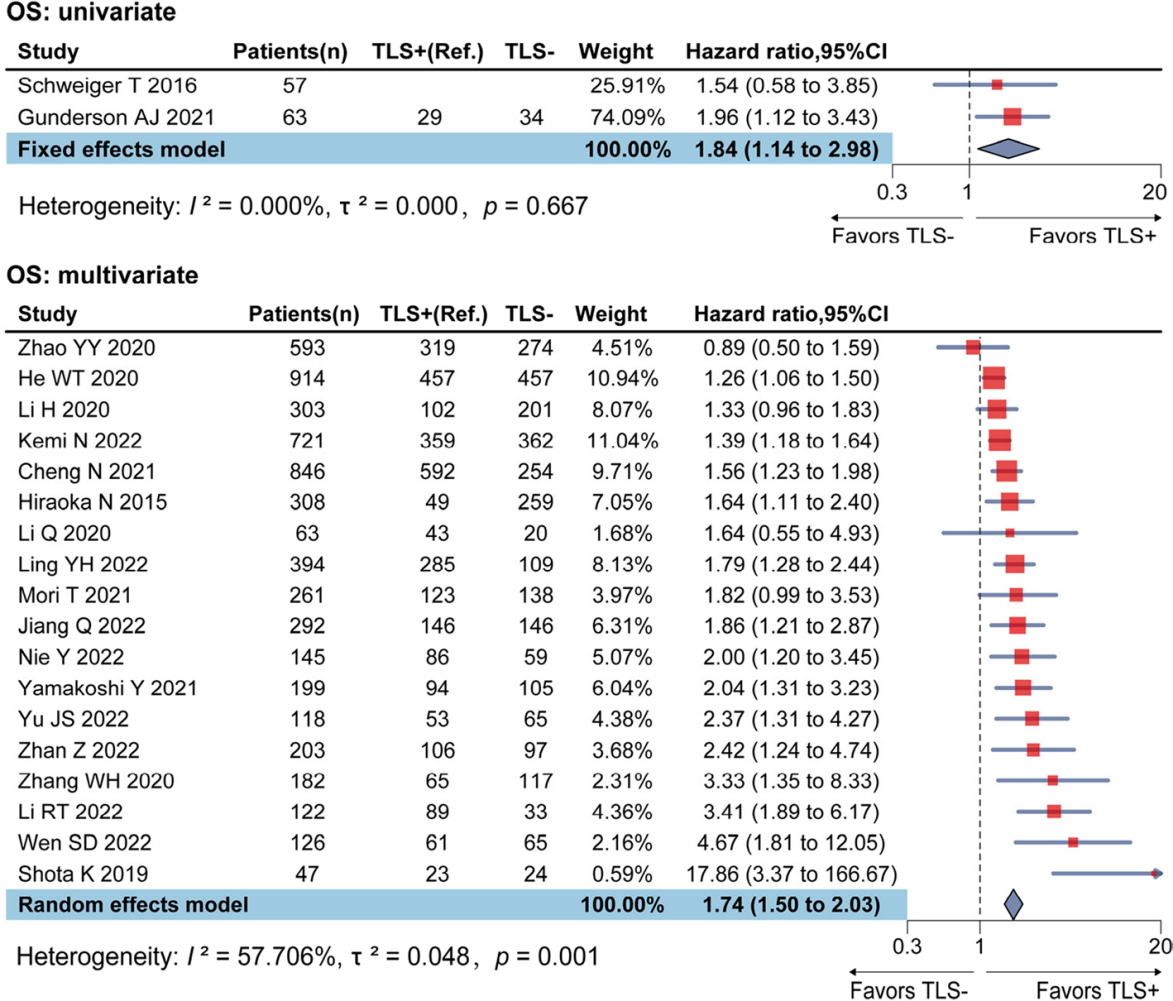
Forest plots for the association between TLS and OS in digestive system cancers

Twenty studies reported HR (95% CI) of TLS on OS, among them, only 2 studies reported univariate analysis results, with insignificant heterogeneity (*I*^2^ = 0.00%, *p* = 0.67), and the combined HR for univariate results was 1.84 (95% CI: 1.14–2.98). Studies (n = 18) reported multivariate analysis results showed a high level of heterogeneity (*I*^2^ = 57.71%, *p* < 0.01), random-effects model yielded a statistically significant combined HR of 1.74 (95% CI: 1.50–2.03), suggesting the absence of TLS was associated with compromised OS for digestive system cancer patients in general (Fig. 2, controlled covariates for multivariate analysis were summarized in Table S2 of supplementary material). Sensitivity analysis using leave-one-out strategy revealed ideal robustness for this combined association (see in supplementary material, Figure S1).

Stratified analyses were performed sequentially by using sample size, metastasis, cut-off criteria of TLS, and cancer types. Sample size presented notable influence on the combined HR of OS: compared with studies of smaller sample size (< 200, HR = 2.52, 95% CI: 1.99–3.20), studies of larger sample size (≥ 200) reached a more conservative pooled association (HR = 1.45, 95% CI: 1.31–1.61), although there was no significant difference between tumor types, the combined HR of pancreatic cancer (HR = 3.37, 95% CI: 1.09–10.37) was higher than other malignant tumors (Table 2, Figure S3-6 in supplementary material).

**Table 2:** Stratified analyses for the association between TLS and OS of digestive system cancers by key factors

	Subgroup	Number of studies	Pooled results HR(95%CI)	Heterogeneity test	
				*I*^2^(%)	*p*
Sample size	≥ 200	10	1.34 (1.31–1.61)	25.51	0.21
	< 200	8	2.52 (1.99–3.20)	22.85	0.25
Metastasis	No	8	1.82 (1.34–2.48)	68.981	< 0.01
	Yes	8	1.86 (1.49–2.33)	48.98	0.06
	NA	2	1.44 (1.26–1.65)	0	0.44
Cut-off criteria	Presence vs. Absence	8	1.69 (1.36–2.10)	48.15	0.05
	High vs. Low	10	1.85 (1.45–2.35)	64.64	< 0.01
Types	ESCC	3	1.76 (0.86–3.61)	80.21	< 0.01
	GC	8	1.54 (1.33–1.79)	28.80	0.20
	CRC	1	2.42 (1.24–4.74)	NA	NA
	HCC	3	2.05 (1.08–3.88)	70.89	0.03
	PC	3	3.37 (1.09–10.37)	71.76	0.03

*Abbreviations*: *ESCC* esophageal squamous cell carcinoma, *CRC* colorectal cancer, *GC* gastric cancer, *HCC* hepatocellular carcinoma, *PC* pancreatic cancer, *OS* overall survival, *RFS* relapse-free survival, *DFS* disease-free survival, *TLS* tertiary lymphoid structure, *NA* not available

### TLS with RFS and DFS of digestive system cancers

**Fig. 3:**
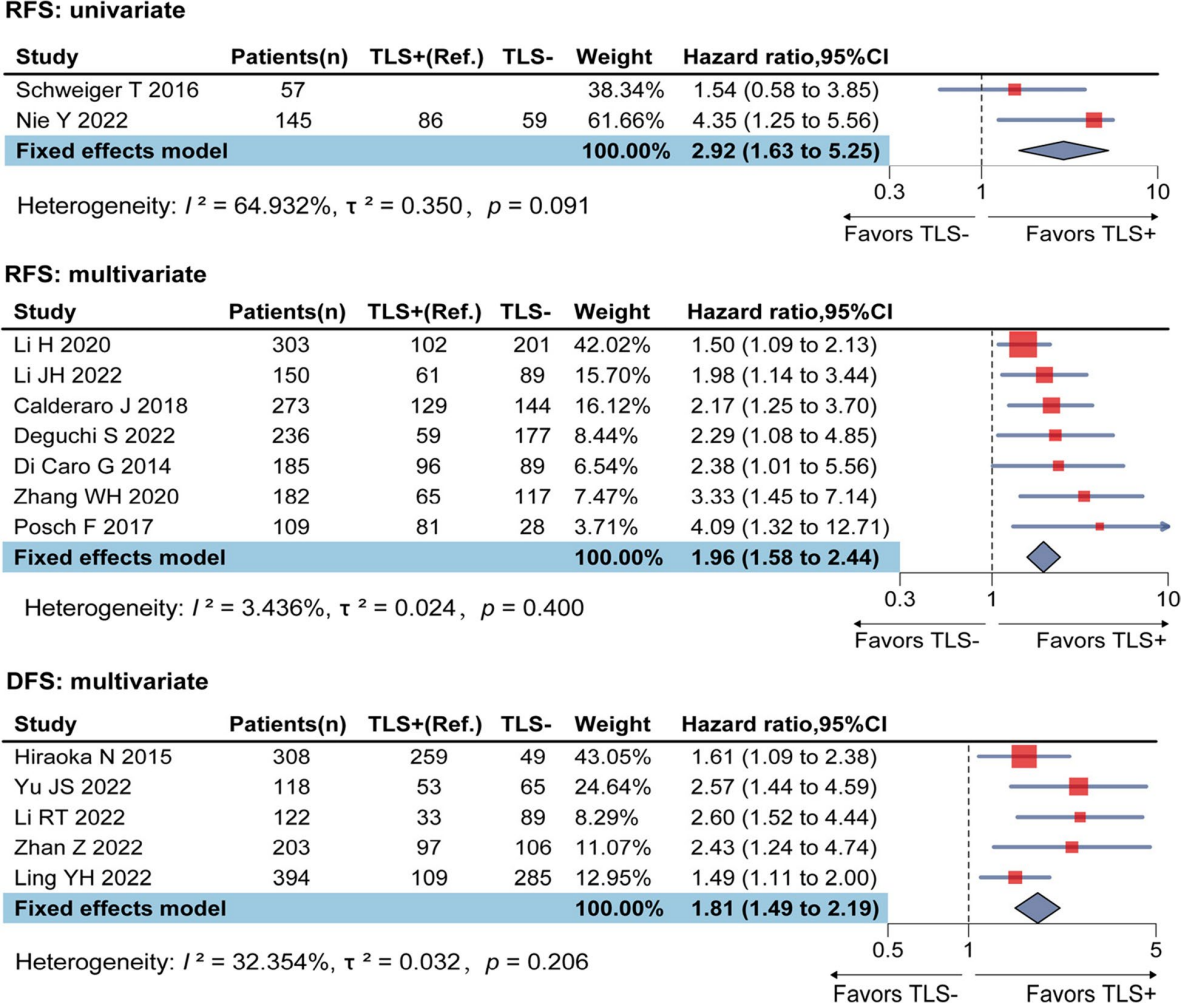
Forest plots for the associations between TLS and RFS, DFS in digestive system cancers

Nine studies reported HR of TLS on RFS: 2 studies reported univariate analysis results, and 7 studies reported multivariate analysis results. The univariate analysis results showed insignificant heterogeneity (*I*^2^ = 64.93%, *p* = 0.09), with a combined HR of 2.92 (95% CI: 1.63–5.25). Heterogeneity for multivariate analysis results were also insignificant (*I*^2^ = 3.44%, *p* = 0.40), fixed-effects model reached a pooled HR of 1.96 (95% CI: 1.58–2.44) (Fig. 3, controlled covariates for multivariate analysis were summarized in Table S2 of supplementary material). Five studies reported HR of TLS on DFS, all used multivariate analysis, with insignificant heterogeneity (*I*^2^ = 32.35%, *p* = 0.21), the pooled HR was statistically significant (HR = 1.81, 95% CI: 1.49–2.19) (Fig. 3, controlled covariates for multivariate analysis were summarized in Table S2 of supplementary material). Sensitivity analysis revealed ideal robustness for included studies of RFS and DFS (see in supplementary material, Figure S2).

### Publication bias

We used funnel plots with Egger’s and Begg’s tests to detect potential publication bias. Funnel plots of OS and RFS showed that the included studies were not perfectly symmetrical, with significant publication bias as suggested by Begg’s and Egger’s tests (see in supplementary material, Figure S7-8). Funnel plot of DFS showed that the included studies were approximately symmetrical, with insignificant publication bias (see in supplementary material, Figure S9).

## Discussion

In this systematic review and meta-analysis, we investigated the association between TLS and the prognosis of patients with digestive system tumors. The synthesized results indicate that based on currently available evidence, the absence of TLS was associated with significantly inferior OS, DFS, and RFS in patients of digestive system cancers. Besides, strength of the association between TLS and OS varied by tumor types, stronger in patients with pancreatic cancer. These important findings suggest that TLS probably plays a role in the prognosis of digestive system tumors, especially for pancreatic cancer.

A successful antitumor immune response requires the presence, activation, and costimulation of all lymphoid components of the immune system, including CD8 + T cells, CD4 + T cells, B cells, and innate lymphocytes. TLS represents a well-organized cluster of tumor-infiltrating lymphocytes and elicits an advanced immune response [52]. Studies have shown that in colorectal cancer, TLS cooperates with tumor-infiltrating T lymphocytes for a coordinated antitumor immune response and predicts a better prognosis [40]. TLS was associated with increased intra-tumoral CD3 + , CD8 + , CD20 + , decreased infiltration of Foxp3 + and CD68 + cells, and predicted better prognosis in early-stage hepatocellular carcinoma [35]. However, studies have also shown that TLS may promote the development of tumors: in a mouse model, TLS that developed in the inflamed liver during hepatitis provided a growth environment for malignant progenitor hepatocytes and was associated with an increased risk of late recurrence and decreased survival [21]. Therefore, it may be reasonable to speculate that inflammation and infection-induced TLS functions differently from cancer-induced TLS, or only intra-tumor TLS is a significant part of the antitumor immune response.

The prognostic propensity of TLS in digestive system cancers has clinical significance. On one hand, for doctors, the detection of TLS may help them preliminarily evaluate mortality risk of the patients. On the other hand, considering the nature of TLS, use of lymphoid chemokines and their drivers may help induce TLS neogenesis, a promising direction for cancer treatment. It has already been possible to induce local TLS in mouse models [10, 53–55]. In gastric adenomas, homeostatic chemokines (including CXCL13, CCLL9 and CCL21) were associated with the formation of TLS [54]. In experimental breast cancer and pancreatic neuroendocrine tumor models, the combination of anti-angiogenesis and anti-PDL1 therapy increased HEV formation and subsequent TLS formation [55]. Adoptive transfer of Hhep-specific CD4 + T cells to Tfh deficient Bcl6fl/flCd4Cre mice restored antitumor immunity and suggested a therapeutic pathway to treat CRC [53].

The significant association between TLS and survival outcomes did not vary much for studies conducted in patients with or without distant metastases, suggesting that TLS may confer similar survival benefit regardless of disease progression. Published studies revealed that TLS was associated with better survival for metastatic patients of lung cancer, ovarian cancer, and cutaneous melanoma [56–58]. Perhaps because the presence of TLS in metastatic sites is a critical factor for tumor-infiltrating lymphocyte levels [56], a crucial factor in anti-tumor immunity which relates to improved prognosis in a variety of solid cancer types [59]. Another important finding would be that the association between TLS and OS was significantly stronger in pancreatic cancer patients. Over 90% pancreatic cancer cases are pancreatic adenocarcinoma (PDAC), one study demonstrated that TLS is almost universal in human PDAC tissue [50], and TLS locates at intratumoral tissue was generally associated with better survival [60]. Moreover, two included studies estimated the association between TLS density and prognosis of gastrointestinal tumors with positive findings [37, 48], suggesting that not only the presence, but also the density of TLS in tumor tissue should be concerned.

This meta-analysis is an exhaustive attempt in synthesizing currently available evidence on the association between TLS and survival in patients with digestive system tumors. The major findings of this study can be consolidated by meticulous literature screening process and strict quality evaluation standard. However, limited number of original studies included, especially for RFS and DFS, hampered effective analysis in discussing possible sources of heterogeneity. Besides, as most of the included studies were originated from 2 Asian countries (China and Japan), the combined estimations of this meta-analysis may suffer from selection bias, more studies should be done in other countries or continents.

## Conclusion

In this meta-analysis, we systematically evaluated the prognostic significance of TLS in digestive system cancers. We found that the absence of TLS was in general associated with worse survival, especially for pancreatic cancer patients. More original studies need to be done, particularly in patients outside Asian countries, to further corroborate this suspected beneficial role of TLS in survival of gastrointestinal tumors.

### Supplementary Information

**Additional file 1:** **Supplementary Materials.** **Table S1.** Quality assessment details for included studies. **Table S2.** Summary of controlled covariates in multivariate analyses. **Figure S1.** Sensitivity analysis for TLS with OS in digestive system cancers patients. **Figure S2.** Sensitivity analysis for TLS with RFS and DFS in digestive system cancers patients. **Figure S3.** Forest plots for stratified analysis by sample size in the association between TLS and OS of digestive system cancers. **Figure S4.** Forest plots for stratified analysis by metastasis state in the association between TLS and OS of digestive system cancers. **Figure S5.** Forest plots for stratified analysis by cut-off criteria in the association between TLS and OS of digestive system cancers. **Figure S6.** Forest plots for stratified analysis by tumor types in the association between TLS and OS of digestive system cancers. **Figure S7.** Funnel plot for publication bias of included studies on the association between TLS and the OS of digestive system cancers. **Figure S8.** Funnel plot for publication bias of included studies on the association between TLS and the RFS of digestive system cancers. **Figure S9.** Funnel plot for publication bias of included studies on the association between TLS and the DFS of digestive system cancers.

## Data Availability

The data that support the findings of this study are available from the corresponding author upon reasonable request.
