# Supplementary material for: Prognostic value of tertiary lymphoid structures (TLS) in digestive system cancers: a systematic review and meta-analysis
Source: BMC Cancer. 2023 Dec 18;23:1248. doi: 10.1186/s12885-023-11738-w (PMC10729333; doi:10.1186/s12885-023-11738-w)
Supplement: Supplementary file 1 — Additional file 1: Supplementary Materials. Table S1. Quality assessment details for included studies. Table S2. Summary of controlled covariates in multivariate analyses. Figure S1. Sensitivity analysis for TLS with OS in digestive system cancers patients. Figure S2. Sensitivity analysis for TLS with RFS and DFS in digestive system cancers patients. Figure S3. Forest plots for stratified analysis by sample size in the association between TLS and OS of digestive system cancers. Figure S4. Forest plots for stratified analysis by metastasis state in the association between TLS and OS of digestive system cancers. Figure S5. Forest plots for stratified analysis by cut-off criteria in the association between TLS and OS of digestive system cancers. Figure S6. Forest plots for stratified analysis by tumor types in the association between TLS and OS of digestive system cancers. Figure S7. Funnel plot for publication bias of included studies on the association between TLS and the OS of digestive system cancers. Figure S8. Funnel plot for publication bias of included studies on the association between TLS and the RFS of digestive system cancers. Figure S9. Funnel plot for publication bias of included studies on the association between TLS and the DFS of digestive system cancers. [file 12885_2023_11738_MOESM1_ESM.docx]

**Supplementary Materials**

Inventory of Supplementary materials

1.Supplementary Retrieval Methods…………………………………………Pages 2

2.Supplementary Tables..……………………………………..…………….Pages 3-5

3.Supplementary Figures……………………………………………….….Pages 6-10

**Supplementary Retrieval Methods**

[“malignanc” OR “tumor*” OR “cancer*” OR “carcinoma*” OR “neoplasm*” OR “neoplasia*”]

[ “digestive system” OR “digestive tract” OR “alimentary system” OR “biliary tract” OR “bile duct” OR “gastrointestinal” OR “gastric” OR “stomach” OR “esophageal” OR “esophagus” OR “esophageal squamous cell carcinoma” OR “Intestinal” OR “Intestines” OR “colorectal” OR “Liver” OR “Hepatic” OR “Hepatocellular” OR “liver cell adenoma*” OR “Pancreatic” OR “Pancreas” OR “Peritoneal”]

[“Tertiary Lymphoid Structure*” OR “Lymphoid Structures, Tertiary” OR “Tertiary Lymphoid Organ*” OR “Tertiary Lymphoid Tissue*” OR “Ectopic Lymphoid-Like Structure*” OR “ectopic lymphoid organ*” OR “Ectopic Lymphoid Follicle*” OR “Ectopic Lymphoid Formation*” OR “Ectopic Lymph Node*”]

[“prognosis” OR “prognostic” OR “survival” OR “outcome”]

**Supplementary Tables**

**Table S1** Quality assessment details for included studies.

|  | Study Participation | Study Attrition | Prognostic Factor Measurement | Outcome Measurement | Study Confounding | Statistical Analysis and Reporting |
| --- | --- | --- | --- | --- | --- | --- |
| Deguchi S 2022 | low | low | low | Moderate | Moderate | Moderate |
| Li RT 2022 | low | low | low | low | low | low |
| Ling YH 2022 | low | low | low | low | Moderate | low |
| Zhao YY 2020 | low | low | Moderate | low | Moderate | low |
| Cheng N 2021 | low | low | low | low | low | low |
| He WT 2020 | low | low | Moderate | low | low | low |
| Jiang Q 2022 | low | low | low | low | low | low |
| Kemi N 2022 | low | low | Moderate | low | Moderate | low |
| Li Q 2020 | low | low | Moderate | low | Moderate | low |
| Sakimura C 2017 | low | low | low | low | low | low |
| Yamakoshi Y 2021 | low | low | low | low | low | low |
| Zhan Z 2022 | low | low | low | low | low | low |
| Posch F 2017 | low | low | low | low | low | low |
| Di Caro G 2014 | low | low | low | Moderate | Moderate | low |
| Schweiger T 2016 | Moderate | low | low | low | low | low |
| Calderaro J 2018 | low | Moderate | low | low | low | low |
| Li JH 2022 | low | low | low | Moderate | low | low |
| Li H 2020 | low | low | low | low | low | low |
| Nie Y 2022 | low | Moderate | low | low | Moderate | low |
| Wen SD 2022 | low | low | low | low | low | low |
| Hiraoka N 2015 | low | low | low | low | low | low |
| Shota K 2019 | Moderate | low | low | low | low | low |
| Zhang WH 2020 | low | low | low | low | Moderate | low |
| Gunderson AJ 2021 | Moderate | low | low | low | Moderate | low |
| Mori T 2021 | low | low | low | low | Low | low |
| Yu JS 2022 | low | low | low | low | low | low |

**Table S2 Summary of controlled covariates in multivariate analyses.**

| study | OS | RFS | DFS |
| --- | --- | --- | --- |
| Zhao YY 2020 | Size, Depth, Differentiation, Lymphovascular invasion, Lymph node metastasis, INF, TILs |  |  |
| Li H 2020 | HBV DNA, HCV infection, Tumor size, Tumor number, Macrovascular invasion | HBV DNA, Ascites, AFP, Tumor size, Tumor number, Macrovascular invasion, Microvascular invasion, TNM, BCLC |  |
| Kemi N 2022 | Year of diagnosis, Center, Age, Sex, Tumor stage, Lauren classification, Perioperative chemotherapy, Radical resection. |  |  |
| Cheng N 2021 | pTNM stage, Lymphovascular invasion, Perineural invasion, Histologic differentiation, Lauren classification, WHO classification, TILs |  |  |
| Hiraoka N 2015 | Tumour size, Pathologic node status, Pathologic metastasis status, Histological grade, Tumour margin status, Nerve plexus invasion, Lymphatic invasion, Venous invasion, Intrapancreatic neural invasion |  | Tumour size, Pathologic tumour status, Pathologic node status, Pathologic metastasis status, Histological grade, Nerve plexus invasion, Lymphatic invasion, Venous invasion, Intrapancreatic neural invasion |
| Li Q 2020 | Tumor size, Histological grade, Tumor thrombus, Lymphatic metastasis, pTN stage, TILs, gcCD8+ TILs |  |  |
| Ling YH 2022 | Age, pN stage |  | pT stage, pN stage |
| Jiang Q 2022 | Age, Gender, LVI, Grade, HP infection, Lauren classification, AJCC stage |  |  |
| Nie Y 2022 | BCLC,HBsAg,Cirrhosis,AFP |  |  |
| Yamakoshi Y 2021 | pT category, pN category, Histological type, Lymphatic invasion, NLR |  |  |
| Zhan Z 2022 | TNM stage |  | TNM stage |
| He WT 2020 | Age, Tumor size, Vessel invasion, Histological grade, pTN, WHO subtypes |  |  |
| Li RT 2022 | Tumor stage, Lymph node metastasis, Histologic grade |  | Tumor stage, Lymph node metastasis, Histologic grade |
| Wen SD 2022 | ECOG PS, BCLC, TB, GGT, NLR |  |  |
| Shota K 2019 | Location, Tumor size, Pathologic node status |  |  |
| Li JH 2022 |  | AST, Lymph, ALB, Tumour capsule, Child grade, BCLC Stage |  |
| Calderaro J 2018 |  | Age, Sex, BCLC stage, AFP , Etiology (HBV), Etiology (NASH), Tumor size, Satellite Nodules, Macrovascular invasion, Macrotrabecular-massive, Subtype |  |
| Deguchi S 2022 |  | CD20, CD8 |  |
| Di Caro G 2014 |  | Local invasion, Tumor cell type |  |
| Zhang WH 2020 | TNM stage, WHO classification | AJCC8th TNM stage, WHO classification, Vascular invasion |  |
| Posch F 2017 |  | Age, ECOG performance status, UICC tumor stage, Adjuvant chemotherapy |  |
| Mori T 2021 | pStage, Venous invasion, CD103, CD8 |  |  |
| Yu JS 2022 | CEA, CA199, Chemotherapy, Tumor size, Stage |  | CEA, CA199, Chemotherapy, Tumor size, Stage |

Acronym list: HBV: hepatitis B virus; HCV: hepatitis C virus; AFP: alpha-fetoprotein; INF: infiltrative; LVI: lymphovascular invasion; TILs: tumor infiltrating lymphocytes; TB: total Bilirubin; GGT: gamma glutamyl transpeptidase; NLR: neutrophil-to-lymphocyte ratio; TNM: tumor-nodes-metastasis; ECOG PS: Eastern Cooperative Oncology Group performance status; AJCC: American Joint Committee on Cancer; BCLC: Barcelona Clinic Liver Cancer; CEA: carcinoembryonic antigen; CA199: Carbohydrate antigen199.

**Supplementary Figures**


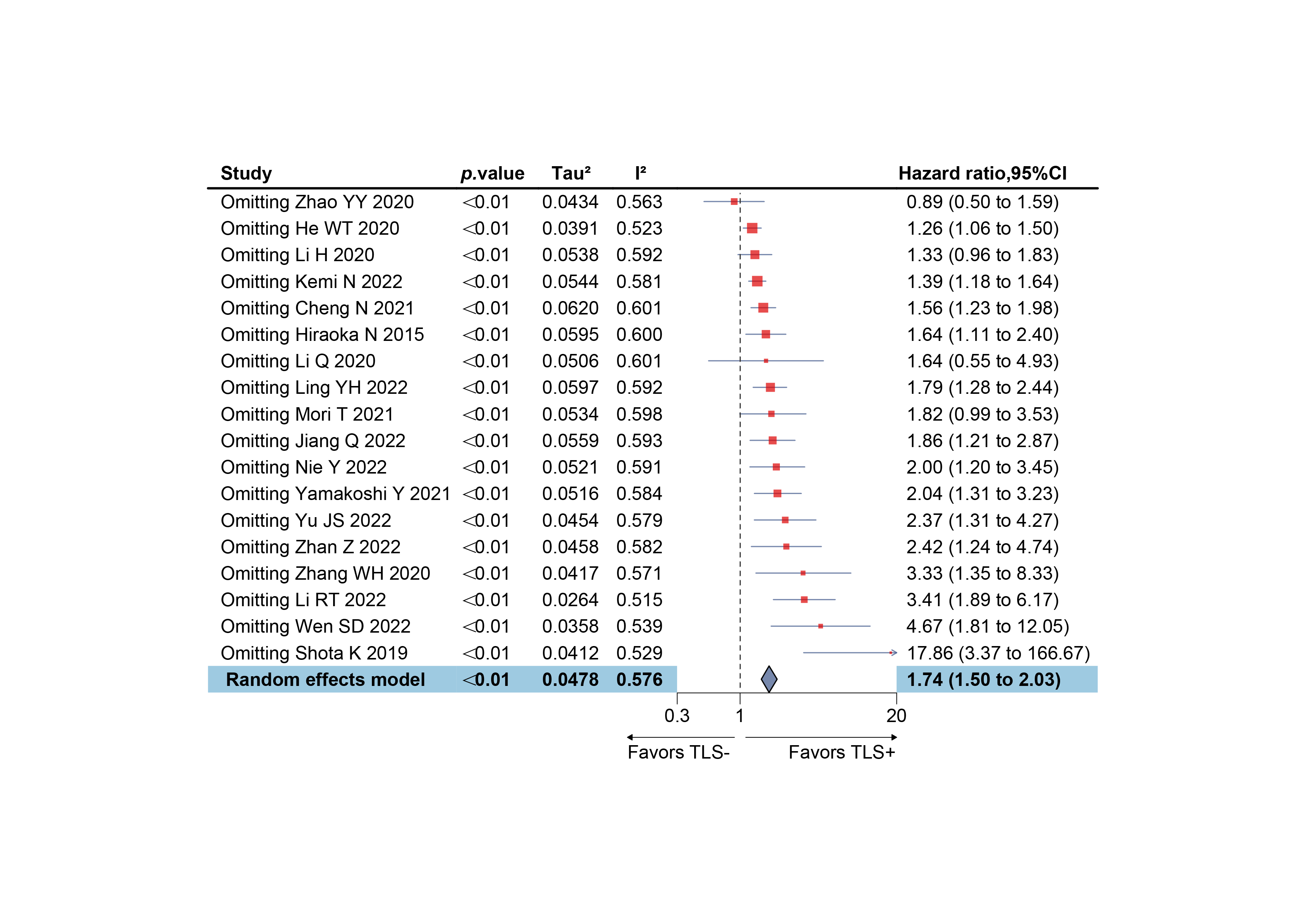


**Figure** **S1** Sensitivity analysis for TLS with OS in digestive system cancers patients.


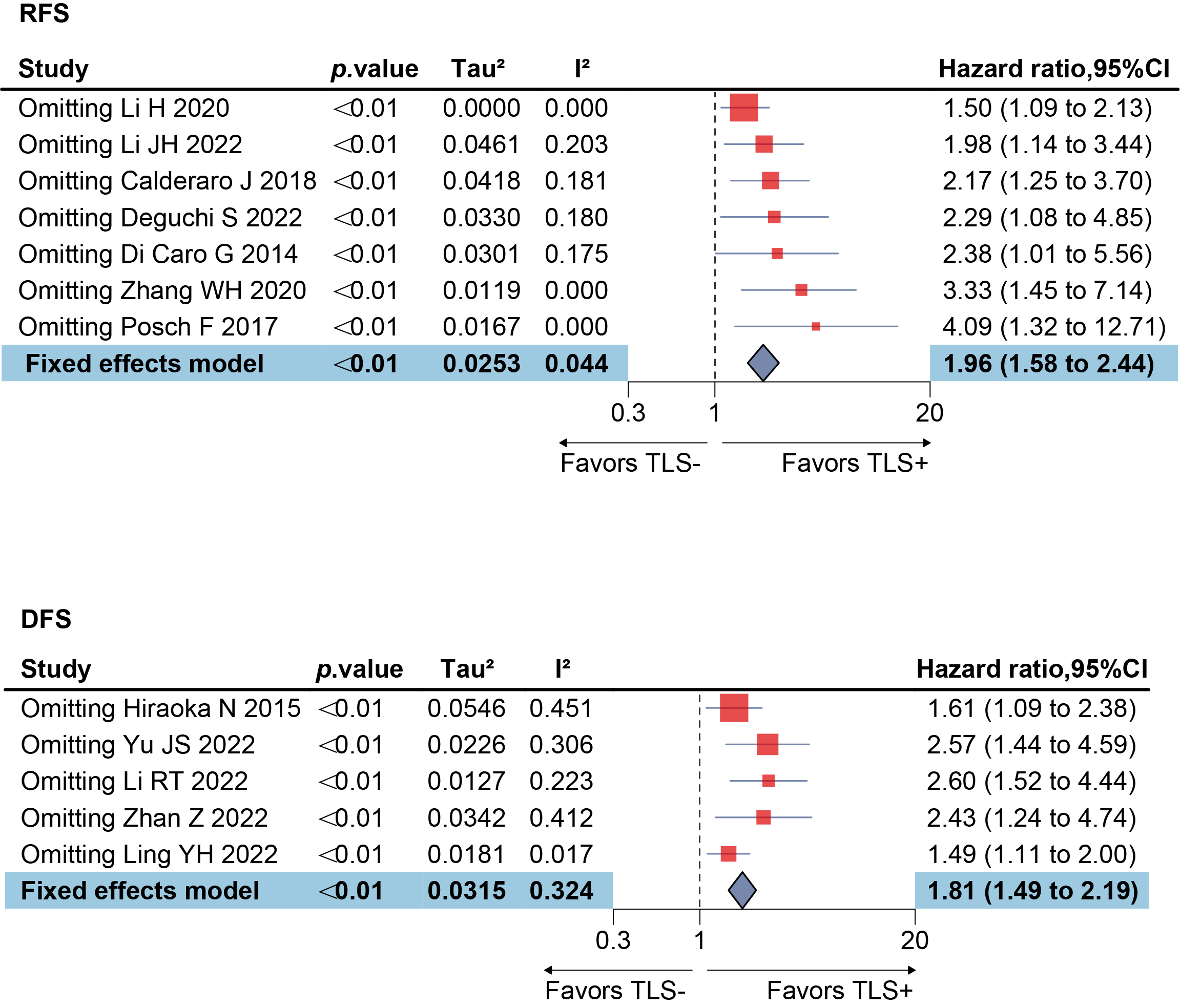


**Figure S2** Sensitivity analysis for TLS with RFS and DFS in digestive system cancers patients.


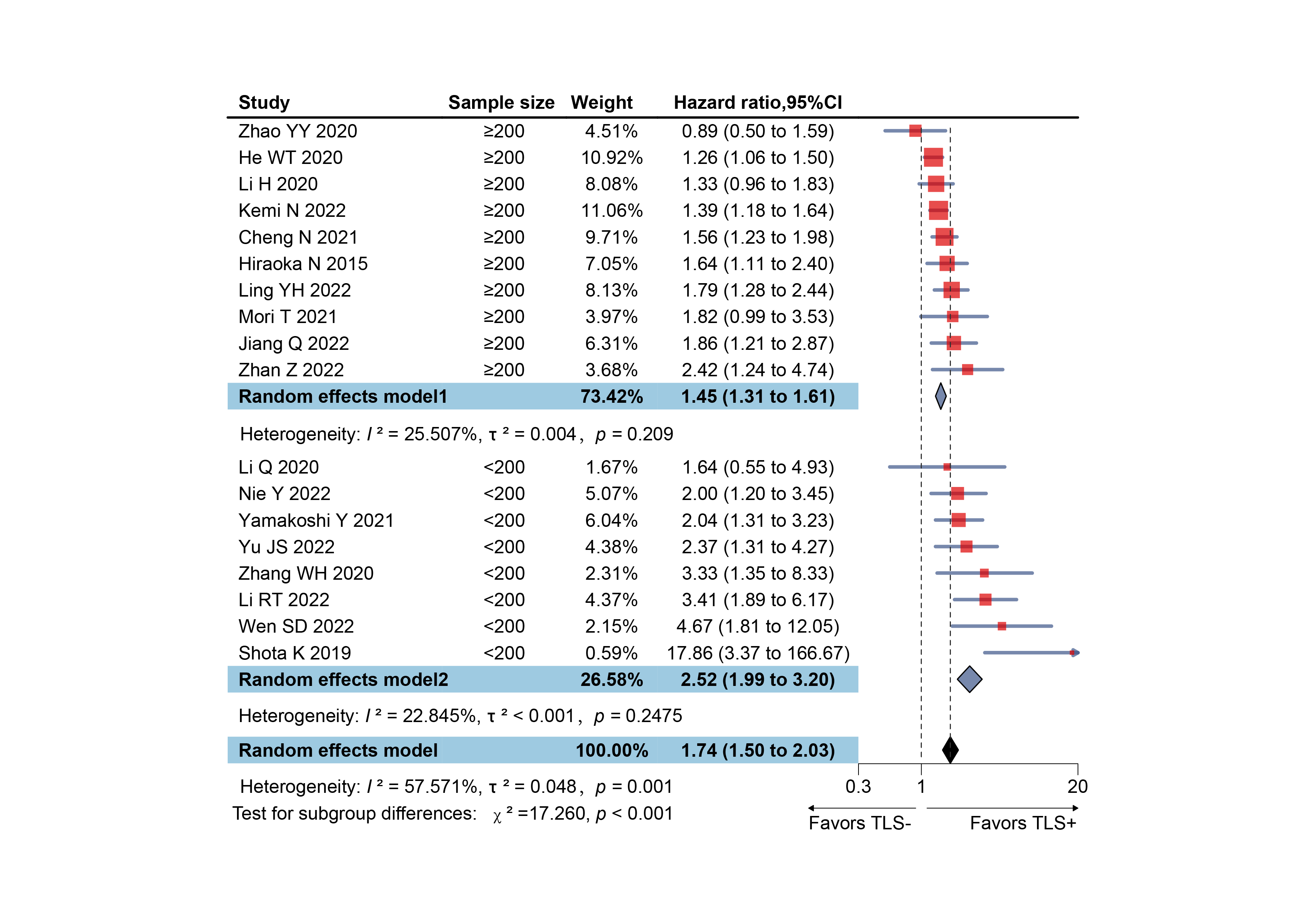


**Figure S3** Forest plots for stratified analysis by sample size in the association between TLS and OS of digestive system cancers.


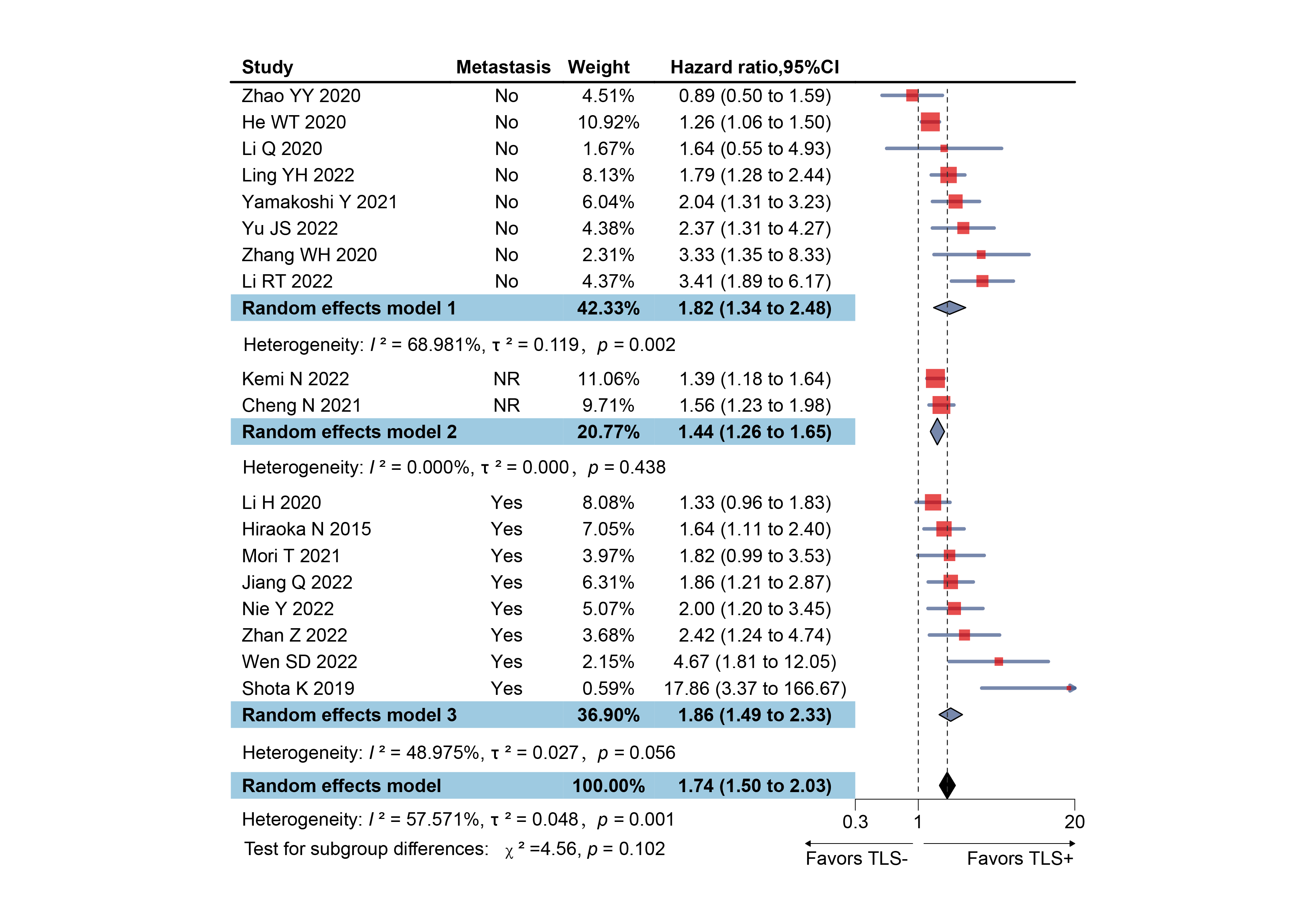


**Figure S4**  Forest plots for stratified analysis by metastasis state in the association between TLS and OS of digestive system cancers.


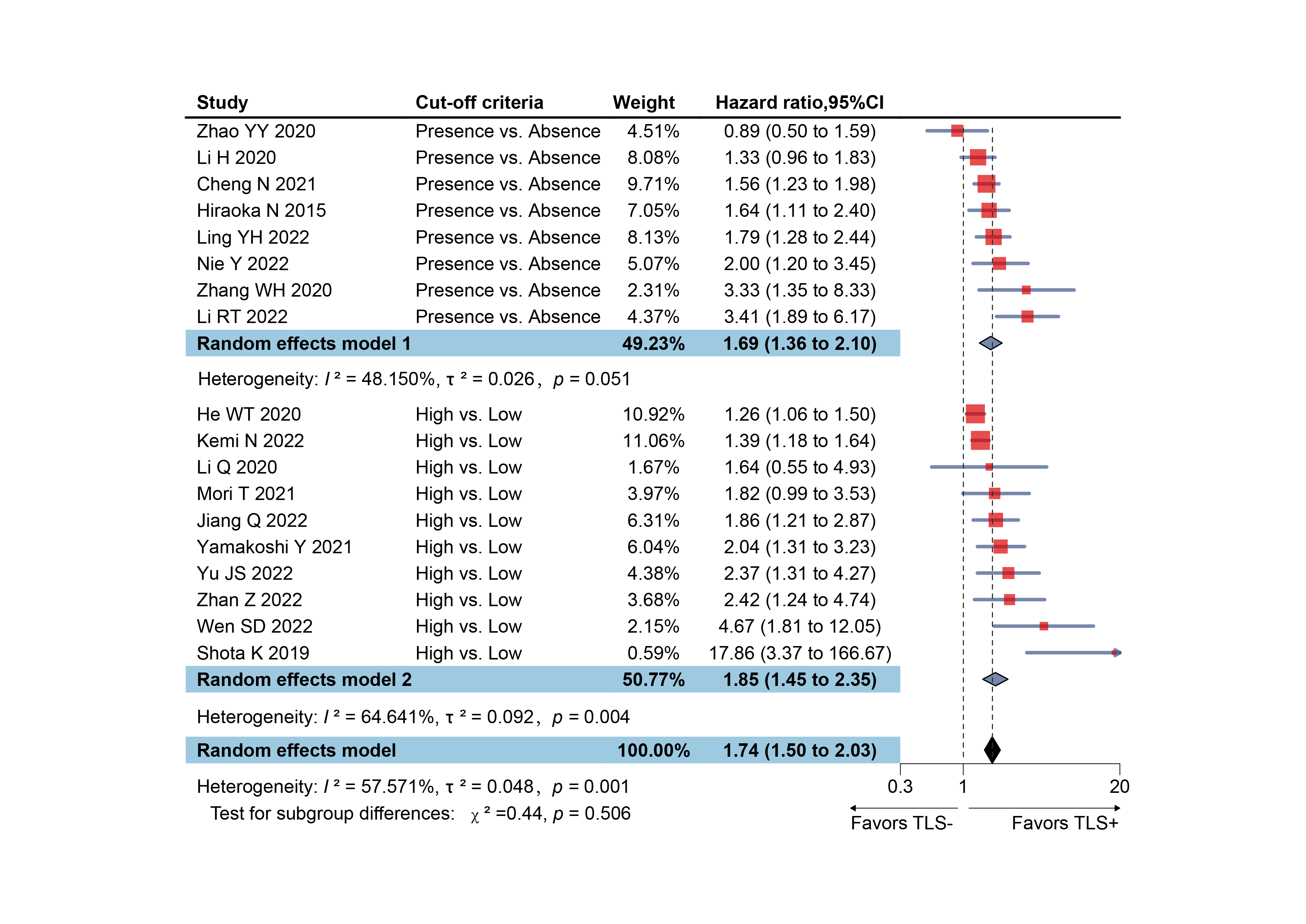


**Figure S5**  Forest plots for stratified analysis by cut-off criteria in the association between TLS and OS of digestive system cancers.


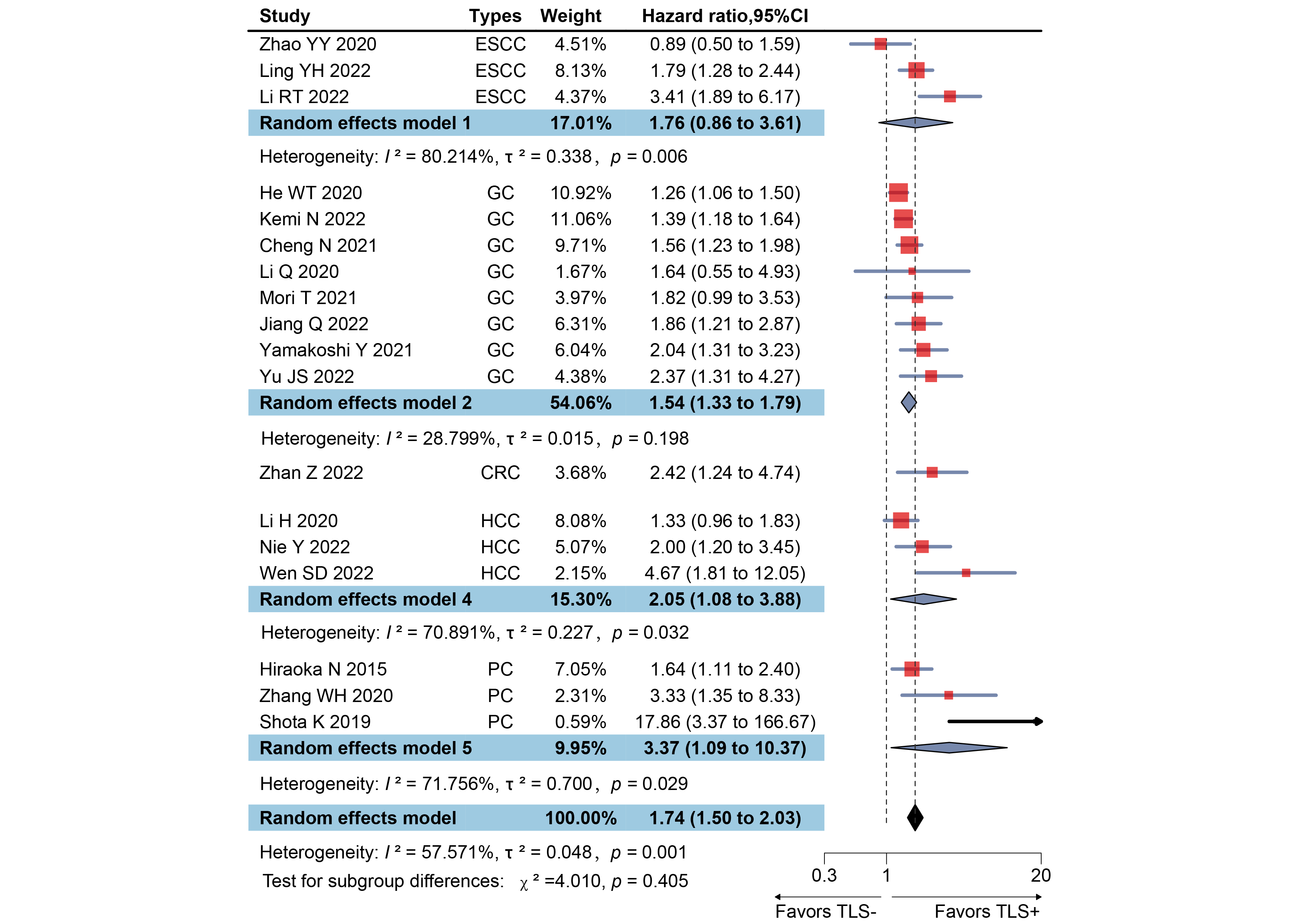


**Figure S6** Forest plots for stratified analysis by tumor types in the association between TLS and OS of digestive system cancers.


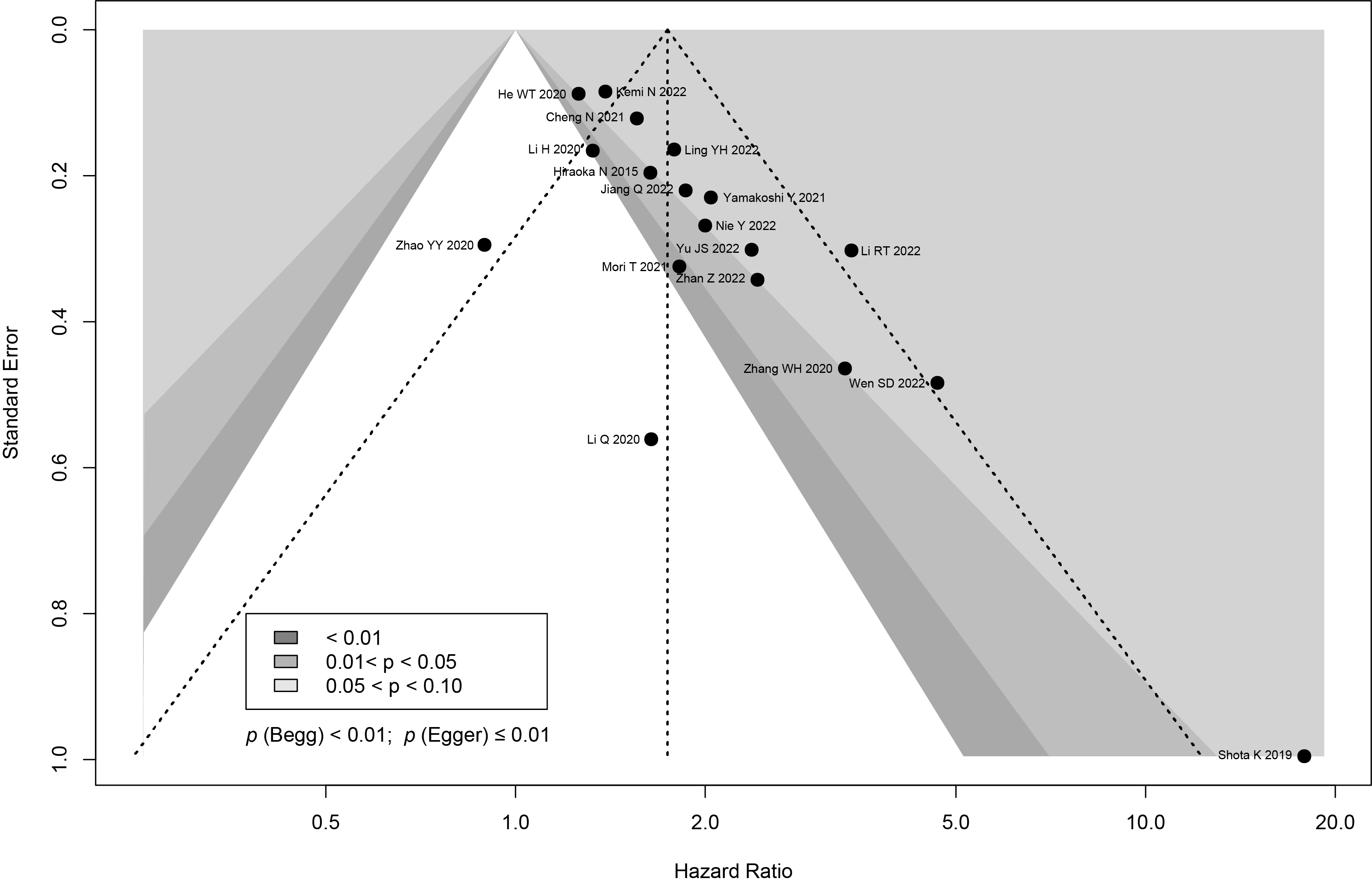


**Figure S7**  Funnel plot for publication bias of included studies on the association between TLS and the OS of digestive system cancers.


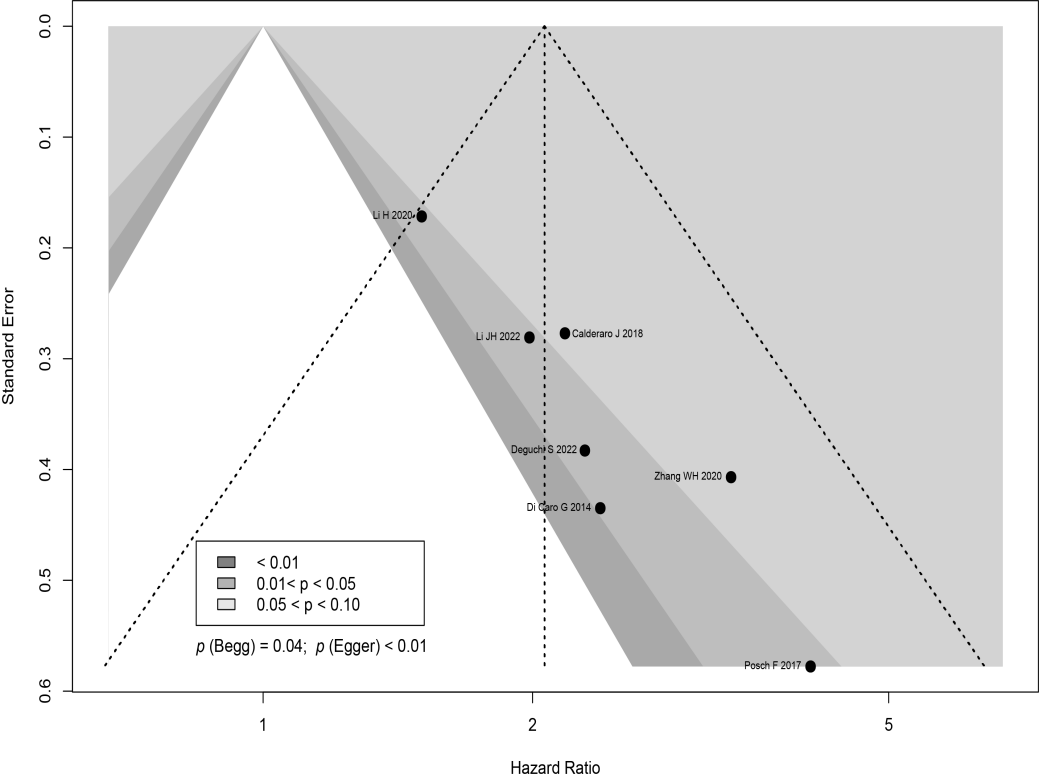


**Figure S8** Funnel plot for publication bias of included studies on the association between TLS and the RFS of digestive system cancers.


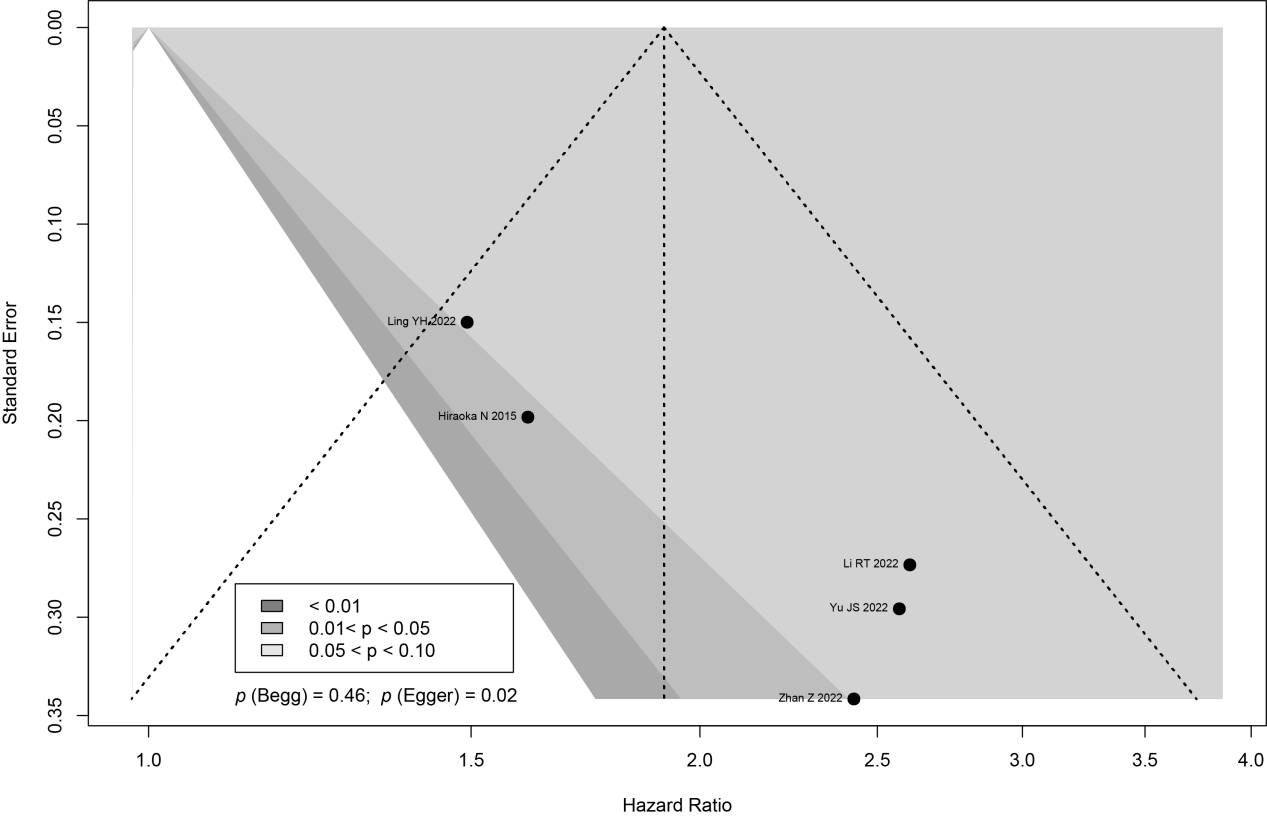


**Figure S9** Funnel plot for publication bias of included studies on the association between TLS and the DFS of digestive system cancers.
